# How to Rank Journals

**DOI:** 10.1371/journal.pone.0149852

**Published:** 2016-03-01

**Authors:** Corey J. A. Bradshaw, Barry W. Brook

**Affiliations:** 1 School of Biological Sciences, The University of Adelaide, Adelaide, South Australia 5005, Australia; 2 School of Biological Sciences, Private Bag 55, University of Tasmania, Hobart 7001, Australia; Universidad de Las Palmas de Gran Canaria, SPAIN

## Abstract

There are now many methods available to assess the relative citation performance of peer-reviewed journals. Regardless of their individual faults and advantages, citation-based metrics are used by researchers to maximize the citation potential of their articles, and by employers to rank academic track records. The absolute value of any particular index is arguably meaningless unless compared to other journals, and different metrics result in divergent rankings. To provide a simple yet more objective way to rank journals within and among disciplines, we developed a *κ*-resampled composite journal rank incorporating five popular citation indices: Impact Factor, Immediacy Index, Source-Normalized Impact Per Paper, SCImago Journal Rank and Google 5-year h-index; this approach provides an index of relative rank uncertainty. We applied the approach to six sample sets of scientific journals from *Ecology* (*n* = 100 journals), *Medicine* (*n* = 100), *Multidisciplinary* (*n* = 50); *Ecology + Multidisciplinary* (*n* = 25), *Obstetrics & Gynaecology* (*n* = 25) and *Marine Biology & Fisheries* (*n* = 25). We then cross-compared the *κ*-resampled ranking for the *Ecology + Multidisciplinary* journal set to the results of a survey of 188 publishing ecologists who were asked to rank the same journals, and found a 0.68–0.84 Spearman’s *ρ* correlation between the two rankings datasets. Our composite index approach therefore approximates relative journal reputation, at least for that discipline. Agglomerative and divisive clustering and multi-dimensional scaling techniques applied to the *Ecology + Multidisciplinary* journal set identified specific clusters of similarly ranked journals, with only *Nature* & *Science* separating out from the others. When comparing a selection of journals within or among disciplines, we recommend collecting multiple citation-based metrics for a sample of relevant and realistic journals to calculate the composite rankings and their relative uncertainty windows.

## Introduction

Love them or loathe them, ‘objective’ metrics designed to measure a peer-reviewed journal’s performance relative to others are here to stay. Journal rankings and scores are, rightly or wrongly [1, 2], used ubiquitously now by academic selection panels to assess applicant track records, by scholars choosing journals to which they will submit their research findings, and by publishing companies seeking to market their journals [3, 4]. The ISI® Impact Factor—calculated as the average number of times the articles from a journal published within the past two years have been cited in the *Journal Citation Reports* year—has, to date, received the most attention [5] and hence, the most criticism [6–8]. Critics have shown that the Impact Factor does not compare well among disciplines [9, 10], it tends to increase over time regardless of journal performance [10, 11], and the methods behind its calculation are not transparent (particularly, what types of articles are counted). This has led to gaming, and as a result, there have been many suggested modifications to the algorithm [1, 2, 12–14]. Nonetheless, the Impact Factor is now entrenched in the psyche of researchers and has arguably changed the dynamic of journal assessment and bibliometrics more than any other single method [15].

Despite its established dominance, the Impact Factor today has many competitors that are all, to some degree, based on citation data. These include *inter alia* ISI’s other metrics such as the five-year average Impact Factor, the Immediacy Index, Cited Half-life, Eigenfactor Score and Article Influence Score, plus Elsevier’s Source-Normalized Impact Per Paper, Impact Per Publication and SCImago Journal Rank, and now Google’s 5-year Hirsch-type [16] index [17] and its median [18]. It has been shown that these different metrics can deliver vastly different rankings for individual journals [18, 19] (and see Supporting Information of this paper). No single metric can be viewed as ideal because some tend to overestimate citations (e.g., Google Scholar) [20], while others underestimate them (e.g., Web of Science- and Scopus-based metrics) [8, 21].

Another issue is the subjective interpretation of journal ‘quality’, for reasons that go well beyond methodological questions of how to combine or relativize citation performance. This is because journal reputation amongst peers, its impact on policy and practice, and the quality of the articles themselves, do not necessarily reflect the number of citations an article or a journal will ultimately receive [22]. It is for these reasons, and the impossible task of choosing a ‘best’ metric, that journal metrics should only ever be considered as indices of relative, average-citation performance from within a discipline-specific or personally selected sample of journals [9]. By itself, the value of a particular journal citation metric is largely meaningless.

Here we describe a simple method to calculate mean metric ranks (and resampled uncertainty bounds) from specific samples of journals, and we provide a simple computer code (R Programming Language script) to do the calculations. Our results provide a more integrated and transparent way for researchers, publishers, and employers to judge a journal of interest relative to any other. We also validate our approach with a journal-ranking survey of perceptions of ‘quality’ from 188 publishing ecologists, both to demonstrate the approach’s utility and to identify its biases.

## Materials and Methods

### Metrics

There are many existing algorithms used to rank journals. The most well-known are the ISI® *Web of Knowledge* (webofknowledge.com) metrics, including Impact Factor (**IF**): the average number of times articles from the journal published in the past two years have been cited in the *Journal Citation Reports* year; 5-year Impact Factor (**IF5**): the average IF over last five years; Immediacy Index (**IM**): the average number of times an article is cited in the year it is published; Citation Half-Life (**HL**): the median age of articles cited by the journal in the *Journal Citation Reports* year; Eigenfactor Score (**EFS**): is based on the number of times articles from the journal published in the past five years have been cited in the *Journal Citation Reports* year, but it also considers which journals have contributed these citations so that highly cited journals will influence the network more than lesser-cited journals. References from one article in a journal to another article from the same journal are removed, so that EFS is not influenced by journal self-citation [23]; and Article Influence Score (**AIS**): calculated by dividing a journal’s EFS by the number of articles in the journal, normalized as a fraction of all articles in all publications [23].

Elsevier also produces three journal metrics based on the Scopus® citation database (www.journalmetrics.com), namely the Source-Normalized Impact Per Paper (**SNIP**): the ratio of a journal's citation count per paper and the citation potential in its subject field (average length of reference lists in a field to determine the probability of being cited to correct for differences between subject fields) [24]; Impact Per Publication (**IPP**): the ratio of citations in a year to papers published the three previous years, divided by the number of papers published in those same years [24]; and SCImago Journal Rank (**SJR**): a measure of scientific influence of scholarly journals that accounts for both the number of citations received by a journal and the importance or prestige of the journals from where such citations come. It is a variant of the eigenvector centrality measure used in network theory [25, 26].

Finally, Google (scholar.google.com) has entered the journal ranking fray with its 5-year Hirsch’s (h) index for journals [16] (**h5**): the largest number *h* such that *h* articles published in the past five years have at least *h* citations each; and median 5-year h-index (**h5m**): the median number of citations for the articles that make up its h5. Here, a journal’s *h*-index (or any other citation-based metric) is entirely unrelated to the original and better known *h-*index of individual researchers [16].

Using metrics up to 2013, we decided to cull our sample of metrics to five of the most disparate, to reduce cross-correlations and redundancy. IF and IF5 were strongly correlated, as were h5 and h5m (S1 Table). Of the three Elsevier metrics (SNIP, IPP and SJR), IPP was the most redundant (S1 Table). We also excluded EFS and AIS given their redundancy relative to IF (webofknowledge.com), as well as HL because that metric did not provide exact values > 10. Although EFS and AIS are arguably more carefully weighted metrics, we used the raw IF in this analysis because of its ubiquity; the three metrics are also highly correlated (Spearman’s *ρ* = 0.83–0.93 based on 85 ‘biology’ journals listed in ISI® Web of Science–data not shown).

Our final list of metrics therefore included IF, IM, SNIP, SJR and h5. For h5, we found a non-random, positive relationship between h5 and the number of articles published that year (Spearman’s *ρ* = 0.52 to 0.75; S1 Table), so we divided h5 by log_10_(*n*) to standardize it per journal. Within each sample of *J* journals, we ranked each journal and each metric from 1 (highest) to *J* (lowest) journals using a simple *rank* function in the R package [27], with ties treated as mean ranks. We provide all raw metric data for each of the sample journals (see below) in the Supporting Information.

### Sample journals

We selected the ‘top’ 100 *Ecology*, 100 *Medicine* and 50 *Multidisciplinary* journals in which biologically themed papers are regularly published (our choice of the journal pool was necessarily somewhat arbitrary, but based on reputation of higher-ranked journals, and historical metrics like IF5). Each journal in each sample was required to have the full set of metrics we examined, including total number of articles published in 2013 derived from ISI (*n*), total number of citations in 2013 derived from ISI (*c*), IF (ISI), IM (ISI), SNIP (Elsevier), SJR (Elsevier), and h5 (Google).

We also generated a fourth example set, consisting of 25 journals that were ecology-specific, but which also included several high-ranking multidisciplinary journals (i.e., a mix of journals from the *Ecology* and *Multidisciplinary* journal sets outlined above). Our rationale was that an ecologist would consider such a range of journals to which she/he might submit a high-quality manuscript. The same idea could apply equally to any other discipline, and so is not dependent on the discipline of ecology *per se*. Given our particular expertise in ecology, we are confident that this list includes a representative and realistic selection of relevant journals in our field (although the final choice of journals is irrelevant to demonstrate our method’s utility). Finally, we included two more discipline-specific sets of 25 journals from *Obstetrics & Gynaecology*, and *Marine Biology & Fisheries*. Our aim here was to examine ranks within a specialist discipline (without multidisciplinary journals) to investigate within-subdiscipline patterns.

### Ranking uncertainty

We did not apply an *a priori* weighting to any of the five metrics included; instead we calculated the mean and standard deviation of each metric’s rank per journal. We calculated a resampled uncertainty interval (i.e., not a true confidence interval because of the finite sample of journals considered) of that mean rank by resampling (function *sample* in the R Programming Language) [27] with replacement a random selection of journals for each of 10,000 iterations [28]. For each journal, we took the 0.025^th^ and the 0.975^th^ quantiles of the resampled ranks as the uncertainty bounds. We also applied a kappa (*κ*) limitation to the resampled selections, whereby we only retained the resampled mean ranks within *κσ* of the overall average mean (here we set *κ* = 2), thereafter recalculating the average and standard deviation of the mean rank, and repeating the process five times. We used this iterative *κσ* ‘clipping’ approach—which is often used in image processing to remove artifacts when stacking sub-frames [29]—to limit the influence of outliers in estimating the range of mean rank across all 10,000 iterations. It is important to understand that the resulting rank uncertainties do not represent an estimate of a true statistical parameter because we are only concerned with how much the relative rank of each journal in the selection performs as journals are included or excluded in the randomly resampled selections. A ‘true’ combined ranking uncertainty is therefore nonsensical because presumably one would never be interested in knowing a journal’s specific rank relative to all other existing journals. In other words, it is a random-sampling procedure only for that sample. We also verified the resampled ranking by calculating a jackknifed estimate of the rank uncertainty using the *jackknife* function in the bootstrap library [30] in R. We provide all the R script necessary to repeat the analysis in the Supporting Information.

### Clustering

To determine whether particular journals within a sample fell into distinct groups, we applied both agglomerative (function: *agnes*) and divisive (function: *diana*) hierarchical clustering (Euclidean metric, complete linkage clustering of standardized metric values) from the cluster library in R [31]. Clustering was based on the same five metrics used in the *κ*-resampled approach described above. To assess the statistical evidence for any group identified, we further applied function *pvclust* from the pvclust library [32] to estimate multi-scale bootstrap resampling probabilities for putative clusters [33]. For further visualization of putative clusters, we also applied principal components analysis to the standardized metrics using the *rda* function in the vegan library [34].

### Survey of ecologists for qualitative validation

We designed an online survey using Google Forms (http://goo.gl/forms/5Kqz8OMtBb) aimed specifically at publishing ecologists of any stage of career. We used targeted email lists and social media (*Twitter*, *Facebook*, *WordPress*) to encourage participation. We removed any entries providing an e-mail address not directly associated with a tertiary academic institution, NGO, government agency or private-sector corporation with research capacity, but retained no personally identifying information. We also included an ecology-specific ‘validation’ question to identify and remove answers from bots and non-ecologists–we also deleted any suspect entries based on the answer to that question. In addition to the journal ranking questions (see below), we asked each participant to provide the stage of their career (*Undergraduate*; *Postgraduate*; *Technical Officer*; *Postdoctoral Fellow*; *Junior Academic*; *Senior Academic*; *Professor*; *Other*), number of peer-reviewed publications published to date (0; 2–5; 6–10; 11–25; 26–50; 51–100; >100); institution type (*University*; *NGO*; *Government*; *Private Sector*); gender, and current country of residence. All participants were aware their responses would be used for research purposes and published.

For each of the 25 ‘ecology sample’ journals, we asked respondents to classify the journal into one of the following categories: 1-*Elite*; 2-*Prestigious*; 3-*Reputable*; 4-*Respectable*; 5-*Other*. Although our meaning was an ordinal scale, we were deliberately vague about how a respondent should classify each journal and interpret the category descriptions, asking them to classify based on instinct and without consulting any specific journal metric. We also did not show them any of our results on the metric-based calculations. Our intention was to have a journal’s reputation—in their opinion—guide their selection. To provide a mean rank of the 25 journals, we calculated the mean and standard deviation of the category values across all (188) vetted respondents. To examine the effect of publication experience on these survey results and their correlation to the compound ranks, we subsetted the survey respondents to those with > 50 published articles and repeated the above analyses.

## Results

### Resampled ranks and clusters

**Fig 1:**
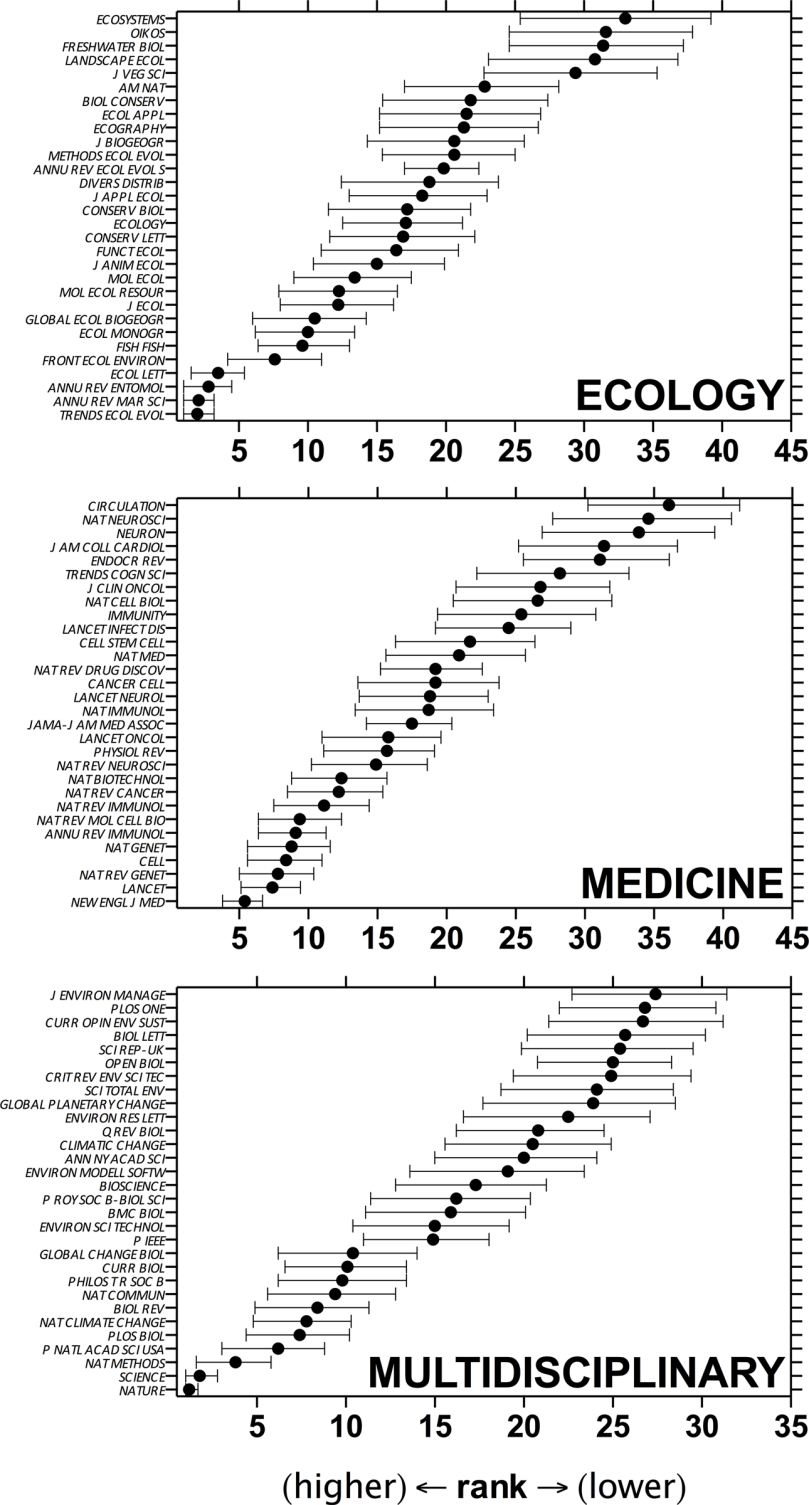
Mean rank (± 95% uncertainty limits) of the top 30 journals for two disparate biological disciplines: *Ecology* and *Medicine*, plus one *Multidisciplinary* theme. Journals are ordered by mean rank of five metrics: IF, IM, SNIP, SJR and h5/log_10_(*n*); statistics were estimated using *κ*-resampling with 10,000 iterations, from a total sample of 100 journals for *Ecology* and *Medicine* and 50 journals for *Multidisciplinary* (see main text for details). Journal abbreviations follow the *Web of Science* standard.

The *κ*-resampling of the mean ranks across metrics revealed an overlapping series of journal ranks per disciplinary sample (Fig 1). Using a mean or median provided similar rankings, but with a few small journal-specific differences (S1 Fig). Ranks were also axiomatically similar based on the jackknife approach (S2 Fig), although the estimated uncertainty was narrower (S3 Fig) given the low number of journal metrics (5) from which to jackknife. For the *Medicine* sample, the greater overall (sample-specific) uncertainty among ranks means that the top-ranked journals in particular are similar, with no clearly dominant journal within the seven or so top-ranked journals within that sample. Given the finite sample of journals, the rank uncertainty windows were necessarily wider in the middle of the range (S4 Fig).

**Fig 2:**
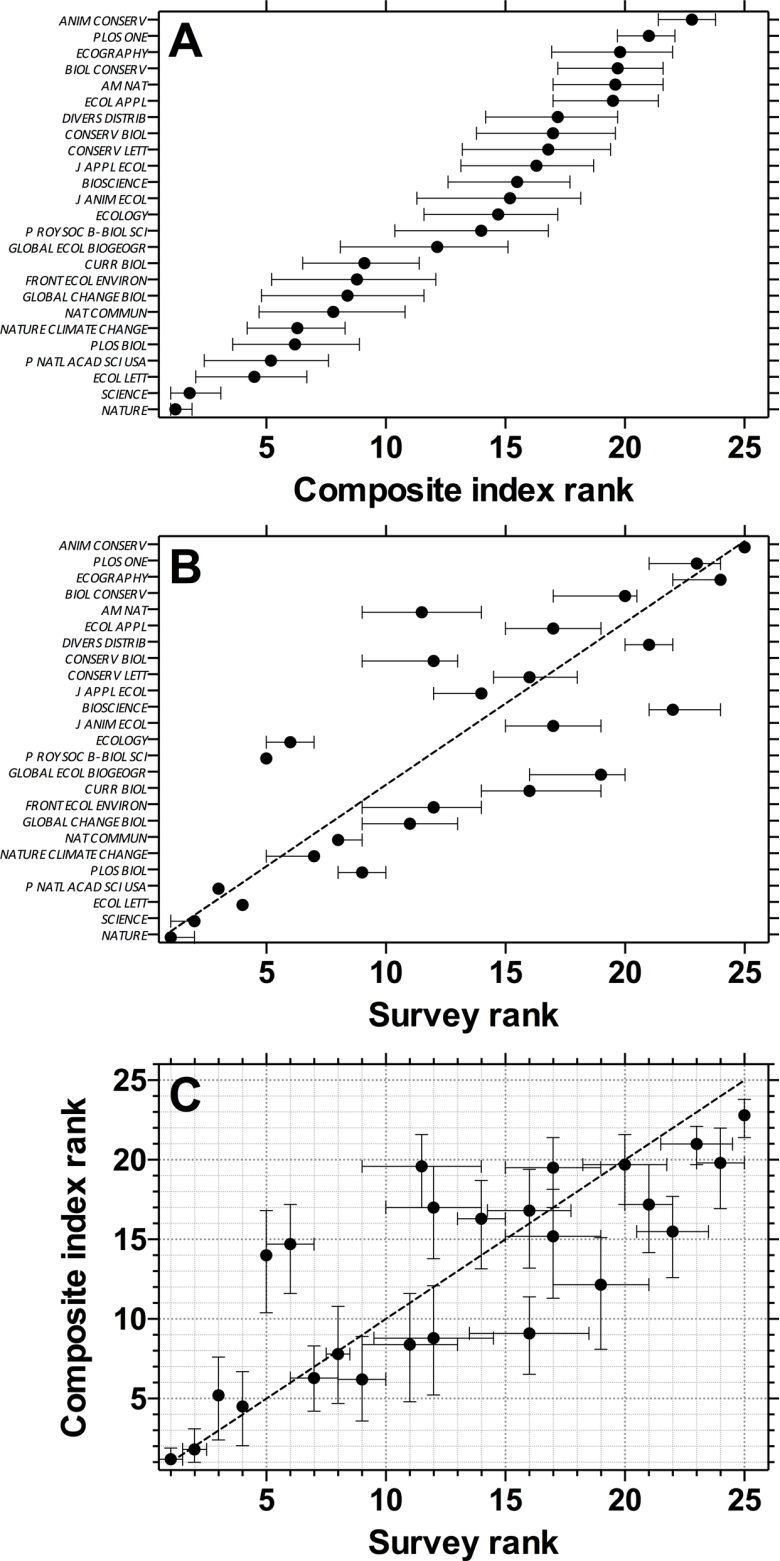
(A) Mean rank (± 95% uncertainty limits via *κ*-resampling with 10,000 iterations) of the top 25 journals within a combined *Ecology* and *Multidisciplinary* theme. Journals are ordered by mean rank of five metrics: IF, IM, SNIP, SJR and h5/log_10_(*n*) (see main text for details). (B) Mean rank (± 1*σ*) of the same journals assessed from a survey of 188 ecologists. Journals above the 1:1 correspondence (45° line) are rated higher by ecologists than their mean metric would indicate, and vice versa. (C) Overall, there was a Spearman’s rank correlation of 0.68–0.84 (median = 0.77; based on 1,000 random uniform resamples of the rank interval) between both rankings. Journal abbreviations follow the *Web of Science* standard.

The ecology-specific sample of 25 journals (*Ecology* + *Multidisciplinary*) yielded another overlapping ranking (Fig 2A) that diverged for some journals from the resampled mean ranks derived from the survey of publishing ecologists (Fig 2B). We had a total of 188 verified-ecologist respondents (49 female, 139 male) from 29 countries (but mainly (69%) from Australia, USA and UK), with an approximately uniform distribution of career stage (postgraduate to professor; S5 Fig). Respondents had a wide range of publication experience (0 to > 100 papers), and were mostly (82%) based in universities (S5 Fig). In particular, the journals *Proceedings of the Royal Society of London B-Biological Sciences*, *Ecology*, *Conservation Biology* and *The American Naturalist* had higher mean reputation scores (and corresponding rank position) than expected from the resampled metric-based mean ranks, and *Current Biology*, *Global Ecology Biogeography* and *BioScience* had lower-than-expected reputation ranks (Fig 2B). All other journals fell near to their expected mean resampled ranks (Fig 2B), with a Spearman’s rank correlation of 0.68–0.84 (median = 0.77; based on 1000 random uniform resamples of the rank uncertainty interval) between the composite metric-based and reputation-based rankings (Fig 2C). Recalculating the rank correlation for only those survey respondents who had published ≥ 50 articles (*n* = 58), the results were nearly identical (Spearman’s rank correlation = 0.67–0.83; median = 0.76), but the resampled mean rank uncertainty intervals were slightly wider (S6 Fig).

**Fig 3:**
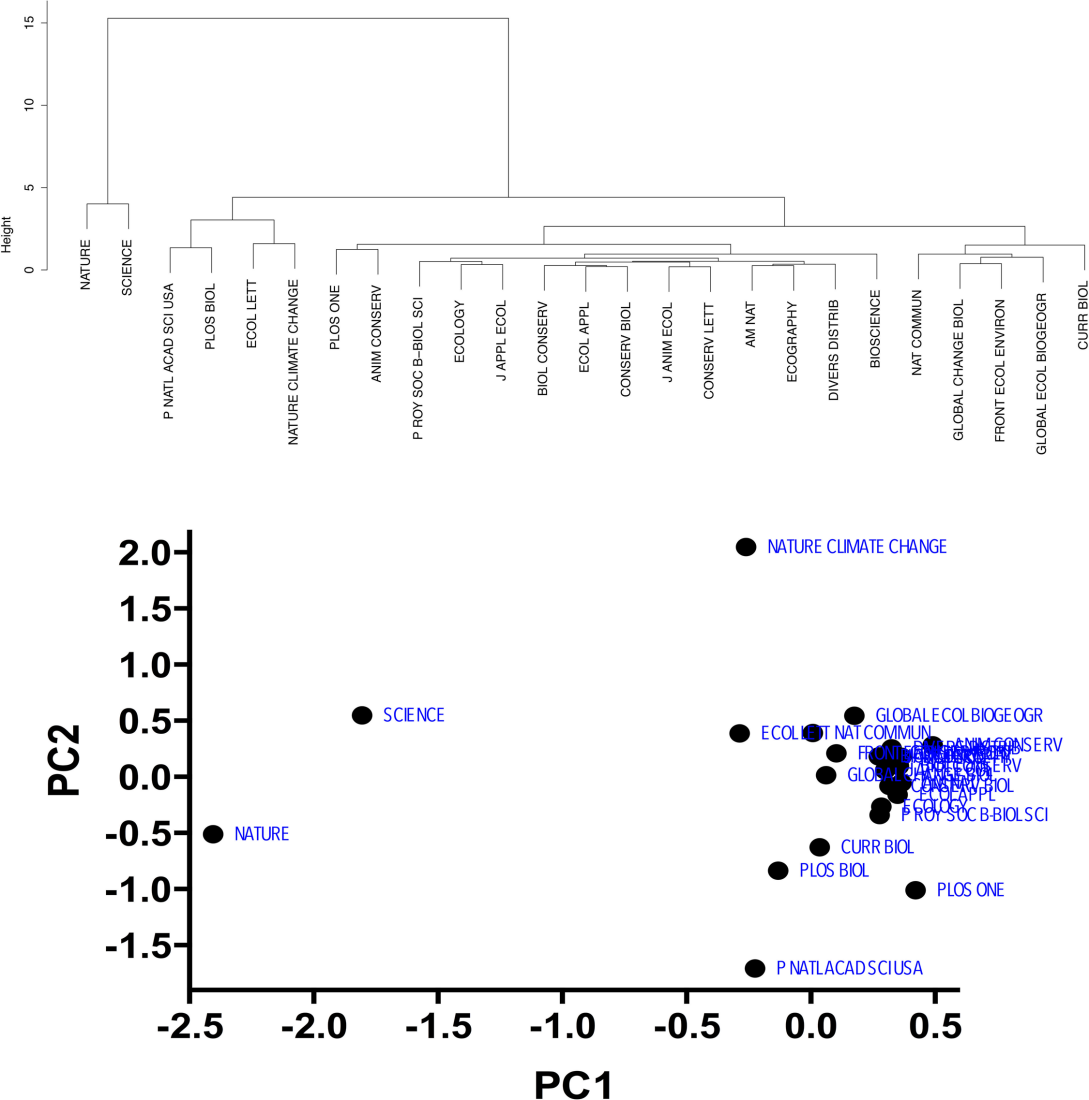
Quality groupings derived from similar mean metrics. Top panel: Agglomerative hierarchical clustering tree for 25 *Ecology* and *Multidisciplinary* journals based on the ranks of five metrics (IF, IM, SNIP, SJR and h5/log_10_(*n*)); calculated as a Euclidean metric, complete linkage clustering of standardized metric values, see main text for details). The clustering methods revealed only two statistically supported groupings: (*i*) *Nature* and *Science* and (*ii*) all remaining 23 journals. Bottom panel: Principal components analysis of the same sample of journals based on their mean ranks from the same five metrics. 95.3% of the variance was explained by the first principal component axis, with only an additional 2.1% explained by the second principal component axis. This confirms the *Science*/*Nature* outliers grouped together using agglomerative and divisive clustering.

Applying the clustering methods to the ecology sample revealed only two statistically supported groupings according to function *pvclust*: (*i*) *Nature* and *Science* and (*ii*) all remaining 23 journals (Fig 3A). The principal components analysis revealed that 95.3% of the variance was explained by the first principal component axis (Fig 3B), with only an additional 2.1% explained by the second principal component axis (Fig 3B), thus confirming the *Science*/*Nature* outliers grouped together using agglomerative and divisive clustering.

**Fig 4:**
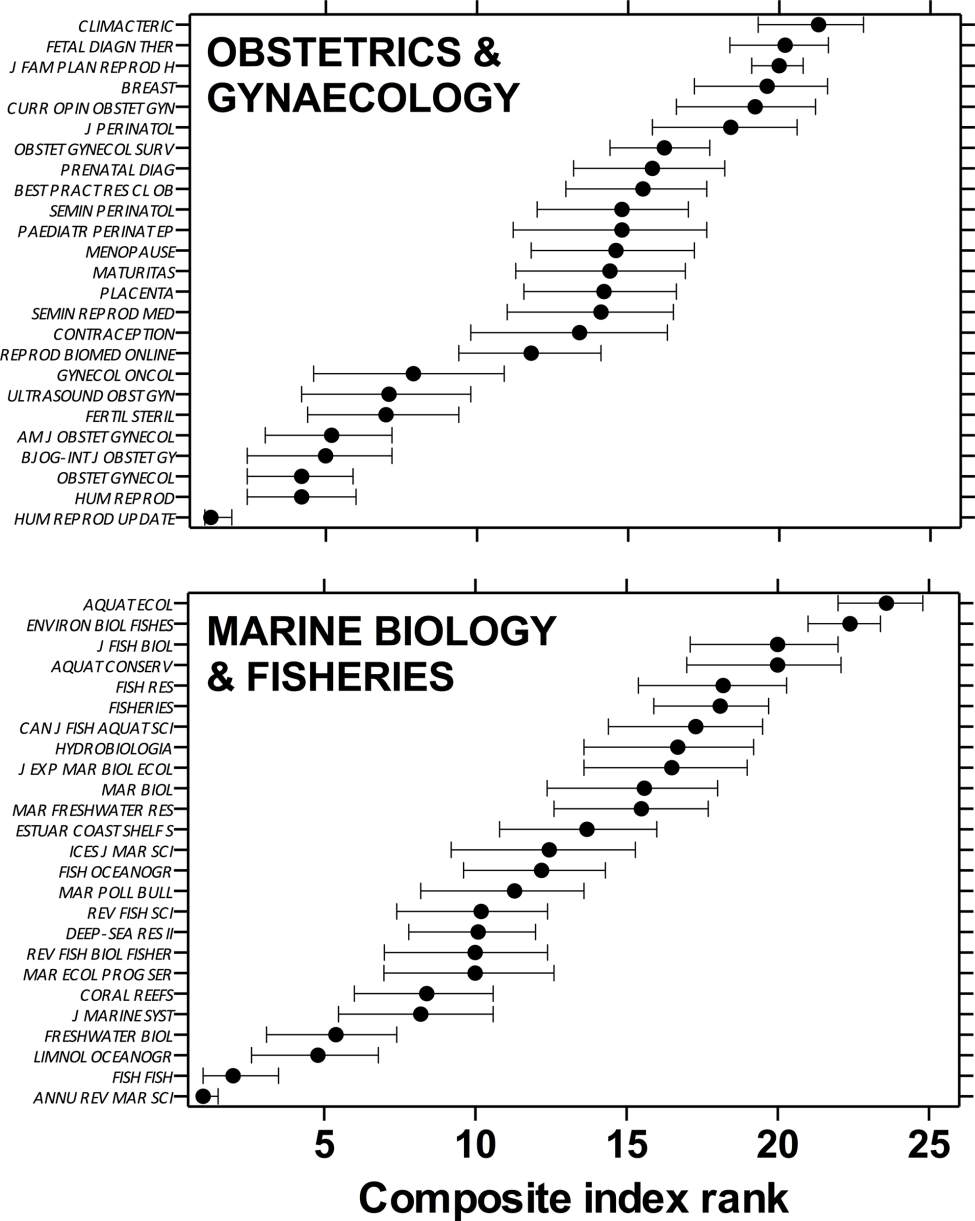
Median rank (± 95% uncertainty limits) of 25 journals within two specialist disciplines: *Obstetrics & Gynaecology* (top panel) and *Marine Biology & Fisheries* (bottom panel). Journals are ordered by mean rank of five metrics: IF, IM, SNIP, SJR and h5/log_10_(*n*); statistics were estimated using *κ*-resampling with 10,000 iterations (see main text for details). Journal abbreviations follow the *Web of Science* standard.

The two specialist discipline samples (*Obstetrics & Gynaecology* and *Marine Biology & Fisheries*) of 25 journals each revealed different patterns of ranking. For the former, there were two clusters of similarly ranked journals from ranks 2–8 and from ranks 10–19, which tended to truncate the spread of ranks across the 25 journals (Fig 4). The ranks of the 25 *Marine Biology & Fisheries* journals were more evenly spread across the spectrum such that there were fewer obvious clusters of similarly ranked journals (Fig 4).

## Discussion

We contend that have designed a more objective and intuitive way than has been previously available to reflect a composite of relative citation-based ranks from within and among specific research disciplines, by using a logical combination of metrics that speak to different aspects of journal caliber. While many other discipline-specific ranking systems have been proposed, such as those based on total downloads [35], author or editor prestige/publishing behavior [36–38], library holdings [37], database coverage [37], and econometric analyses of citations and referencing patterns [39, 40], most suffer from an inability to compare journals across disciplines, rely on overly complex approaches, or make questionable assumptions and subjective choices of the component metrics. Further, while some have also proposed using several citation-based metrics to rank journals (e.g., [41, 42]), our simple approach is the only existing method that explicitly and quantitatively combines the most relevant citation-based metrics into a composite ranking with associated, selection-specific uncertainties.

It was not our intention, however, to discuss the relative merits, shortfalls or quality of the component metrics and the databases from which they are calculated (see [4, 8, 20, 21] for detailed discussion of metric and database issues). Instead, we have provided a method to combine commonly available metrics that most researchers and academic administrators can easily access. Our particular example lists of ranked journals in five biology disciplines (*Ecology*, *Medicine*, *Multidisciplinary*, *Marine Biology & Fisheries* and *Obstetrics & Gynaecology*) represent novel and useful guides for scientists working in these areas (Fig 1 and Fig 4), and we have provided the computer script that anyone can use to construct a personal list of journals to rank in this manner.

Further, by using an independent survey of ecologists from different career stages, institution types, genders and countries of residence, we demonstrated that the perception of relative journal reputation is largely captured by the combined resampled journal rankings. Our sample of respondent ecologists provided a ranking that agreed well with the combined resampled citation-based rankings with a Spearman’s coefficient of 0.68–0.84. This compares favorably to a previous validation survey of journal reputation for physicians, where R^2^ were between 0.62 (practitioners) and 0.83 (researchers) [22] compared to Impact Factors. It is likely that our survey results are not entirely independent of citation metrics because researchers might be subconsciously influenced by them when responding. Regardless of some inevitable circularity, it is instructive that citation-based and reputational survey rankings largely agreed, despite some discipline-specific outliers. Outlier journals themselves might be of interest to researchers to identify an aspect of journal ‘quality’ that is less dependent on citations than what existing metrics currently provide.

Others have attempted to compare the citation performance of individuals, fields and institutions across multiple disciplines, such as dividing citation-based performance by the total number of citations within a specific discipline [43], comparing individual performance to discipline-specific *h*-index confidence intervals [44], standardizing based on citation distribution functions per discipline [45], or comparing multidisciplinary groups using clustering-based bibliometric maps [46]. While some of these techniques could potentially be applied to journal metrics, we are confident that our nonparametric ranking of journals from within any selection represents one of the simplest and most intuitive ways to compare journals across disciplines.

There are of course many considerations authors must canvass when choosing where to submit their papers [15]. In terms of maximizing citation impact, we recommend that researchers consider collecting multiple citation-based metrics for a sample of relevant and realistic journals (i.e., in which it is plausible the manuscript could be published)—say for example, 3–10 journals—and calculate the combined rankings and their uncertainty as we have demonstrated here (see R code provided in S1 File and S2 File for example data). After taking into account target audience, journal scope, acceptance probability, handling time [15], discipline-specific reputation among peers and an appreciation of the journals’ overall ‘quality’ (however objectively or subjectively defined), such relative rankings using combined citation-based metrics might assist researchers to choose the most pertinent journals for manuscript submission. Sample journals could also be a mix of specialist and multidisciplinary/generalist periodicals because our approach takes into account relative rank and not the absolute value of the metrics themselves.

## Supporting Information

S1 File R Programming Language code (text) for repeating analysis. (R)

S2 File Raw 2013 journal citation metric data. (ZIP)

S1 Fig Comparison of resampled mean- and median-based rankings (with uncertainty windows) for each of three samples of journals. Samples include (top) *Ecology*, (middle) *Medicine*, and (bottom) *Multidisciplinary*. Solid grey lines indicate 1:1 correspondence (45° line); dashed black lines indicate least-squares linear fits to the central values.
(TIFF)

S2 Fig Comparison of mean resampled and jackknife rankings for each of six samples of journals. Samples include (A) *Ecology*, (B) *Ecology Sample* (*Ecology* and some *Multidisciplinary* journals), (C) *Medicine*, (D) *Multidisciplinary*, (E) *Obstetrics & Gynaecology*, and (F) *Marine Biology & Fisheries*.
(TIFF)

S3 Fig Compared journal rankings. Resampled (upper panels) versus jackknife (bottom panels) for each of three journal samples (*Ecology*, *Medicine* and *Multidisciplinary*).
(TIFF)

S4 Fig Per-journal rank uncertainty bounds. Rank uncertainty increases nonlinearly through to approximately halfway through the sample, and decrease thereafter (a characteristic of any regression fit), due to the imposed limit of 100 journals in each discipline category (50 for *Multidisciplinary*). Note the greater relative uncertainty for the middle ranks of the *Medicine* discipline.
(TIFF)

S5 Fig Ecology survey respondent characteristics (authors are based in Australia). (TIFF)

S6 Fig *Ecology Sample* journal ranks. (A) Mean rank (± 95% confidence limits via *κ*-resampling with 10,000 iterations) of the top 25 journals within a combined *Ecology* and *Multidisciplinary* theme. Journals are ordered by mean rank of five metrics: IF, IM, SNIP, SJR and h5/log_10_(*n*) (see main text for details). (B) Mean rank (± 1*σ*) of the same journals assessed from a survey of 58 ecologists who had each published ≥ 50 articles. Journals above the 1:1 correspondence (45° line) are rated higher by these ecologists than their mean metric would indicate, and vice versa. (C) Overall, there was a Spearman’s rank correlation of 0.67–0.83 (compared to 0.68–0.84 for the full 188 survey participants); median = 0.76; based on 1,000 random uniform resamples of the rank interval) between both rankings. Journal abbreviations follow the *Web of Science* standard.
(TIFF)

S7 Fig Median rank (± 95% uncertainty limits) of the top 30 journals for two disparate biological disciplines: *Ecology* and *Medicine*, plus one *Multidisciplinary* theme. Journals are ordered by median rank of five metrics: IF, IM, SNIP, SJR and h5/log_10_(*n*); statistics were estimated using *κ*-resampling with 10,000 iterations, from a total sample of 100 journals for *Ecology* and *Medicine* and 50 journals for *Multidisciplinary* (see main text for details). Journal abbreviations follow the *Web of Science* standard.
(TIFF)

S8 Fig Median rank (± 95% uncertainty limits) of 25 journals within two specialist disciplines: *Obstetrics & Gynaecology* (top panel) and *Marine Biology & Fisheries* (bottom panel). Journals are ordered by median rank of five metrics: IF, IM, SNIP, SJR and h5/log_10_(*n*); statistics were estimated using *κ*-resampling with 10,000 iterations (see main text for details). Journal abbreviations follow the *Web of Science* standard.
(TIFF)

S9 Fig Comparison of median resampled and jackknife rankings for each of six samples of journals. Samples include (A) *Ecology*, (B) *Ecology Sample* (*Ecology* and some *Multidisciplinary* journals), (C) *Medicine*, (D) *Multidisciplinary*, (E) *Obstetrics* & *Gynaecology*, and (F) *Marine Biology & Fisheries*.
(TIFF)

S1 Table Spearman’s *ρ* correlation matrix for the individual metrics used to develop the composite ranking. ***n*** = total number of articles published in Journal Citation Reports (JCR; 2013) year; ***cites*** = total number of citations to the journal in the JCR year; ***h5*** = Google 5-year h-index; ***h5m*** = median Google 5-year h-index; ***IF*** = ISI® Impact Factor; ***IF5*** = ISI® 5-year Impact Factor; ***IM*** = ISI® Immediacy Index; ***SNIP*** = Elsevier® Source-Normalized Impact Per Paper; ***IPP*** = Elsevier® Impact Per Publication; ***SJR*** = Elsevier® SCImago Journal Rank.
(DOCX)

## References

[1] Adam D. Citation analysis: the counting house. Nature. 2002; 415(6873):726–9. 11845174 10.1038/415726a

[2] Vanclay J. Impact factor: outdated artefact or stepping-stone to journal certification? Scientometrics. 2012; 92(2):211–38. 10.1007/s11192-011-0561-0

[3] Smith R. Commentary: The power of the unrelenting impact factor—Is it a force for good or harm? International Journal of Epidemiology. 2006; 35(5):1129–30. 10.1093/ije/dyl191 16987843

[4] Jacsó P. The plausibility of computing the h-index of scholarly productivity and impact using reference-enhanced databases. Online Information Review. 2008; 32(2):266–83. 10.1108/14684520810879872

[5] Fersht A. The most influential journals: Impact Factor and Eigenfactor. Proceedings of the National Academy of Sciences of the USA. 2009; 106(17):6883–4. 10.1073/pnas.0903307106 19380731 PMC2678438

[6] PLoS Medicine Editors. The Impact Factor game. PLoS Medicine. 2006; 3(6):e291 10.1371/journal.pmed.0030291 16749869 PMC1475651

[7] Seglen PO. Why the impact factor of journals should not be used for evaluating research. British Medical Journal. 1997; 314(7079):497 10.1136/bmj.314.7079.497 PMC2126010 9056804

[8] Jacsó P. The pros and cons of computing the h-index using Web of Science. Online Information Review. 2008; 32(5):673–88. 10.1108/14684520810914043

[9] Ramírez A, García E, Del Río J. Renormalized Impact Factor. Scientometrics. 2000; 47(1):3–9. 10.1023/A:1005600807292

[10] Althouse BM, West JD, Bergstrom CT, Bergstrom T. Differences in impact factor across fields and over time. Journal of the American Society for Information Science and Technology. 2009; 60(1):27–34. 10.1002/asi.20936

[11] Neff BD, Olden JD. Not so fast: inflation in Impact Factors contributes to apparent improvements in journal quality. BioScience. 2010; 60(6):455–9. 10.1525/bio.2010.60.6.9

[12] Weale AR, Bailey M, Lear PA. The level of non-citation of articles within a journal as a measure of quality: a comparison to the impact factor. BMC Medical Research Methodology. 2004; 4:14 10.1186/1471-2288-4-14 15169549 PMC434502

[13] Zitt M, Small H. Modifying the journal impact factor by fractional citation weighting: The audience factor. Journal of the American Society for Information Science and Technology. 2008; 59(11):1856–60. 10.1002/asi.20880

[14] Pudovkin AI, Garfield E. Rank-normalized impact factor: A way to compare journal performance across subject categories. Proceedings of the American Society for Information Science and Technology. 2004; 41(1):507–15. 10.1002/meet.1450410159

[15] Salinas S, Munch SB. Where should I send it? Optimizing the submission decision process. PLoS ONE. 2015; 10(1):e0115451 10.1371/journal.pone.0115451 25616103 PMC4304711

[16] Hirsch JE. An index to quantify an individual's scientific research output. Proceedings of the National Academy of Sciences of the USA. 2005; 102(46):16569–72. 10.1073/pnas.0507655102 16275915 PMC1283832

[17] Braun T, Glänzel W, Schubert A. A Hirsch-type index for journals. Scientometrics. 2006; 69(1):169–73. 10.1007/s11192-006-0147-4

[18] Delgado-López-Cózar E, Cabezas-Clavijo Á. Ranking journals: could Google Scholar Metrics be an alternative to Journal Citation Reports and Scimago Journal Rank? Learned Publishing. 2013; 26(2):101–13. 10.1087/20130206

[19] Falagas ME, Kouranos VD, Arencibia-Jorge R, Karageorgopoulos DE. Comparison of SCImago journal rank indicator with journal impact factor. The FASEB Journal. 2008; 22(8):2623–8. 10.1096/fj.08-107938 18408168

[20] Jacsó P. The pros and cons of computing the h-index using Google Scholar. Online Information Review. 2008; 32(3):437–52. 10.1108/14684520810889718

[21] Jacsó P. The pros and cons of computing the h-index using Scopus. Online Information Review. 2008; 32(4):524–35. 10.1108/14684520810897403

[22] Saha S, Saint S, Christakis DA. Impact factor: a valid measure of journal quality? Journal of the Medical Library Association. 2003; 91(1):42–6. PubMed PMID: PMC141186. 12572533 PMC141186

[23] Bergstrom CT, West JD, Wiseman MA. The Eigenfactor™ Metrics. The Journal of Neuroscience. 2008; 28(45):11433–4. 10.1523/jneurosci.0003-08.2008 18987179 PMC6671297

[24] Moed HF. Measuring contextual citation impact of scientific journals. Journal of Informetrics. 2010; 4(3):265–77. 10.1016/j.joi.2010.01.002

[25] González-Pereira B, Guerrero-Bote VP, Moya-Anegón F. A new approach to the metric of journals’ scientific prestige: the SJR indicator. Journal of Informetrics. 2010; 4(3):379–91. 10.1016/j.joi.2010.03.002

[26] Guerrero-Bote VP, Moya-Anegón F. A further step forward in measuring journals’ scientific prestige: the SJR2 indicator. Journal of Informetrics. 2012; 6(4):674–88. 10.1016/j.joi.2012.07.001

[27] R Core Team. R: A language and environment for statistical computing Vienna, Austria: R Foundation for Statistical Computing, 2014.

[28] Manly BFJ. Randomization, Bootstrap and Monte Carlo Methods in Biology, Third Edition London: Chapman and Hall/CRC Press; 2006. 480 p.

[29] Lehmann G. Kappa sigma clipping. Insight Journal. 2006; http://hdl.handle.net/1926/367.

[30] Tibshirani R, Leisch F. *bootstrap*: Functions for the Book "An Introduction to the Bootstrap". R package version 20152. 2015:http://CRAN.R-project.org/package=bootstrap.

[31] Maechler M, Rousseeuw P, Struyf A, Hubert M, Hornik K. *cluster*: cluster analysis basics and extensions. R Package version 1153. 2014:http://CRAN.R-project.org/package=cluster.

[32] Suzuki R, Shimodaira H. *pvclust*: hierarchical clustering with p-values via multiscale bootstrap resampling. R package version 12–2. 2011:http://CRAN.R-project.org/package=pvclust.

[33] Shimodaira H. Approximately unbiased tests of regions using multistep-multiscale bootstrap resampling. Annals of Statistics. 2004; 32(6):2616–41. 10.1214/009053604000000823

[34] Oksanen J, Blanchet FG, Kindt R, Legendre P, Minchin PR, O'Hara RB, et al. *vegan*: community ecology package. R package version 20–10. 2013:http://CRAN.R-project.org/package=vegan.

[35] Brown LD. Ranking journals using social science research network downloads. Review of Quantitative Finance and Accounting. 2003; 20(3):291–307. 10.1023/A:1023628613622

[36] Korobkin R. Ranking journals: some thoughts on theory and methodology. Florida State University Law Review. 1999:851–76.

[37] East JW. Ranking journals in the humanities: an Australian case study. Australian Academic and Research Libraries. 2006; 37(1):3–16. 10.1080/00048623.2006.10755319

[38] Pujol F. Ranking journals following a matching model approach: an application to public economics journals. Journal of Public Economic Theory. 2008; 10(1):55–76. 10.1111/j.1467-9779.2008.00351.x

[39] Hudson J. Ranking journals. The Economic Journal. 2013; 123(570):F202–F22. 10.1111/ecoj.12064

[40] Kodrzycki YK, Yu P. New approaches to ranking economics journals. The BE Journal of Economic Analysis and Policy. 2006; 5(1):1935–682.

[41] Bontis N, Serenko A. A follow‐up ranking of academic journals. Journal of Knowledge Management. 2009; 13(1):16–26. 10.1108/13673270910931134

[42] Mingers J, Harzing A-W. Ranking journals in business and management: a statistical analysis of the Harzing data set. European Journal of Information Systems. 2007; 16(4):303–16.

[43] Podlubny I. Comparison of scientific impact expressed by the number of citations in different fields of science. Scientometrics. 64(1):95–9. 10.1007/s11192-005-0240-0

[44] Malesios CC, Psarakis S. Comparison of the h-index for different fields of research using bootstrap methodology. Quality and Quantity. 2012; 48(1):521–45. 10.1007/s11135-012-9785-1

[45] Iglesias JE, Pecharromán C. Scaling the h-index for different scientific ISI fields. Scientometrics. 2007; 73(3):303–20. 10.1007/s11192-007-1805-x

[46] van Raan AFJ. Measurement of central aspects of scientific research: performance, interdisciplinarity, structure. Measurement: Interdisciplinary Research and Perspectives. 2005; 3(1):1–19. 10.1207/s15366359mea0301_1

