# Supplementary figures and images for: How to Rank Journals
Source: PLoS One. 2016 Mar 1;11(3):e0149852. doi: 10.1371/journal.pone.0149852 (PMC4773013; doi:10.1371/journal.pone.0149852)

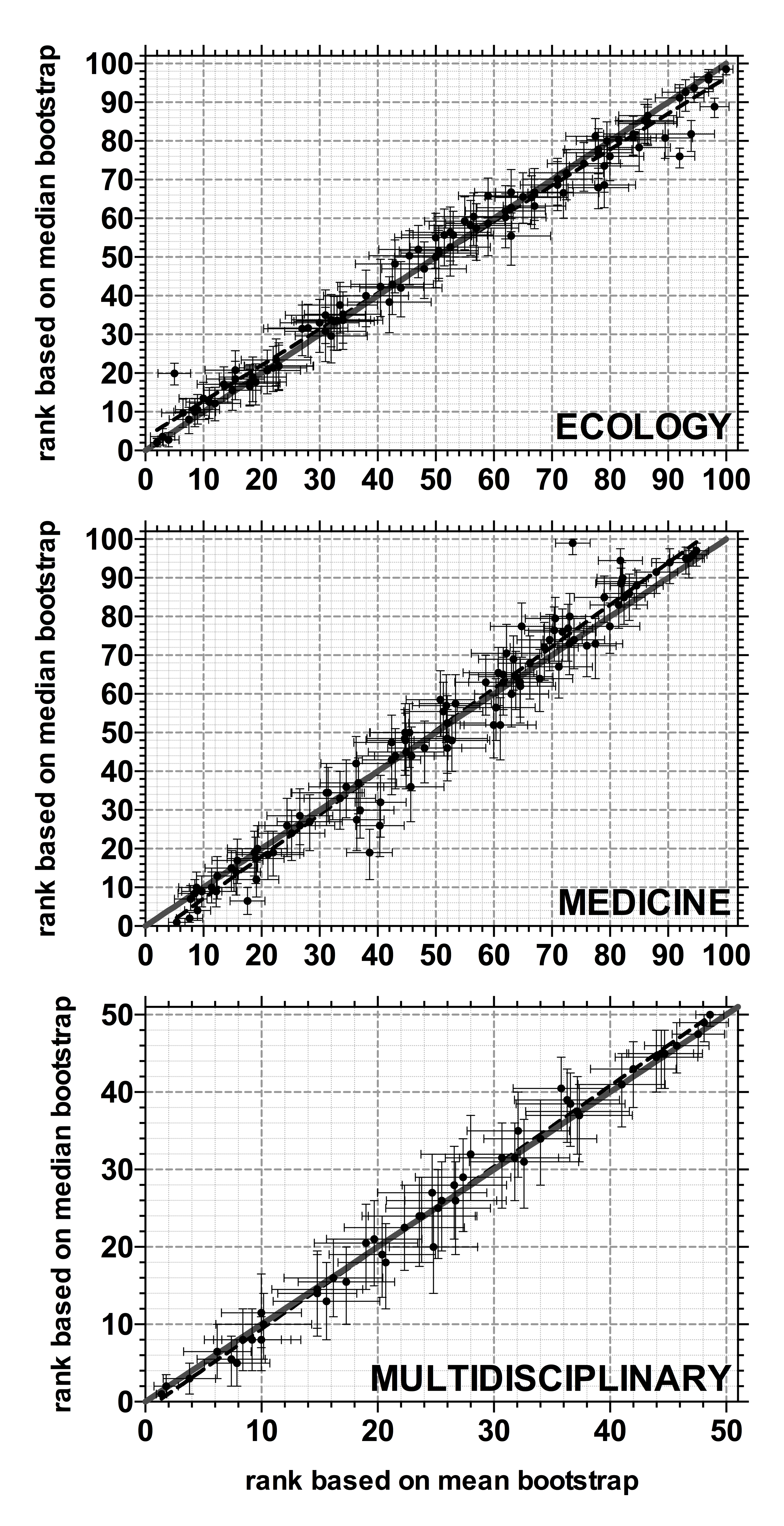

Supplement: S1 Fig — Samples include (top) Ecology, (middle) Medicine, and (bottom) Multidisciplinary. Solid grey lines indicate 1:1 correspondence (45° line); dashed black lines indicate least-squares linear fits to the central values. (TIFF) [file pone.0149852.s003.tiff]

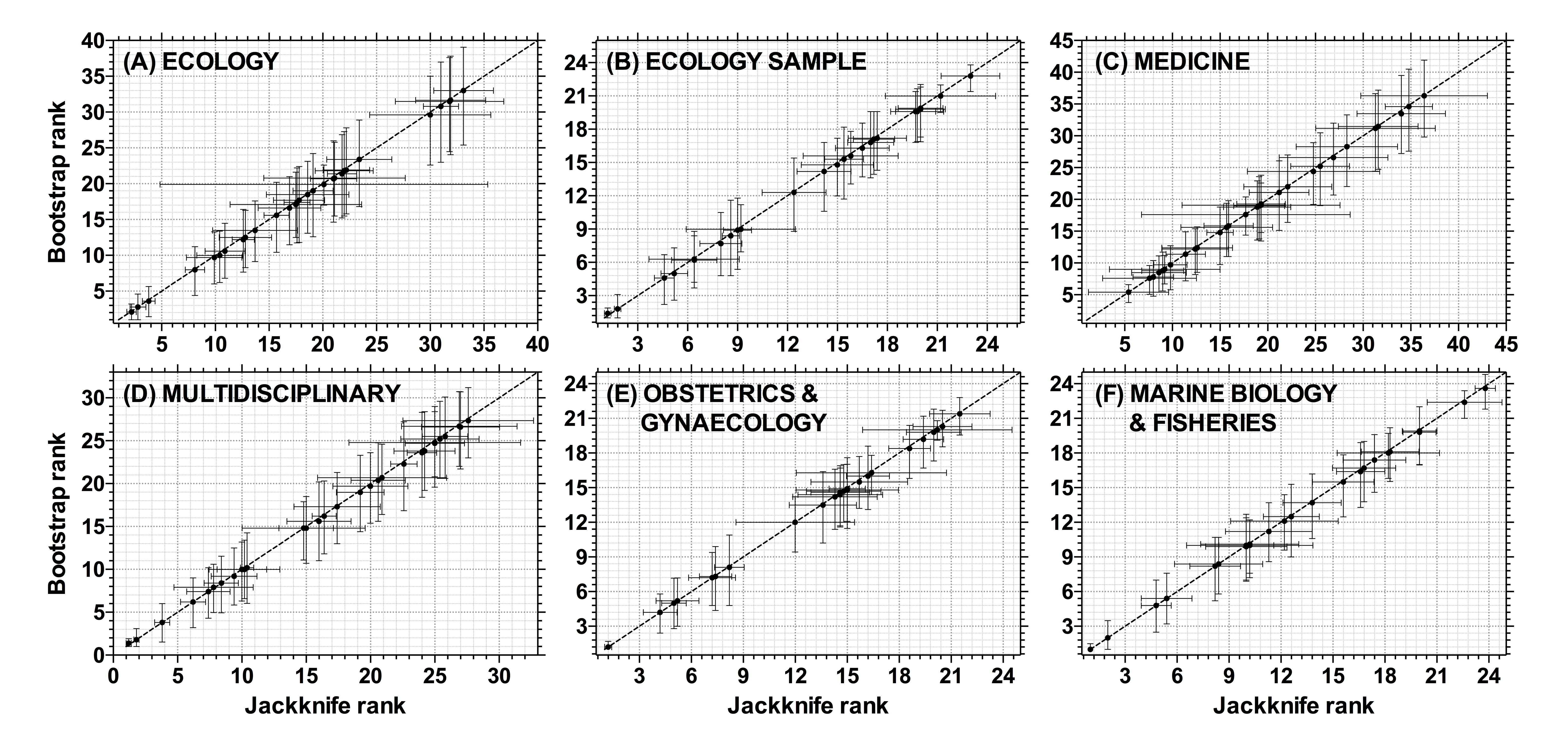

Supplement: S2 Fig — Samples include (A) Ecology, (B) Ecology Sample (Ecology and some Multidisciplinary journals), (C) Medicine, (D) Multidisciplinary, (E) Obstetrics & Gynaecology, and (F) Marine Biology & Fisheries. (TIFF) [file pone.0149852.s004.tiff]

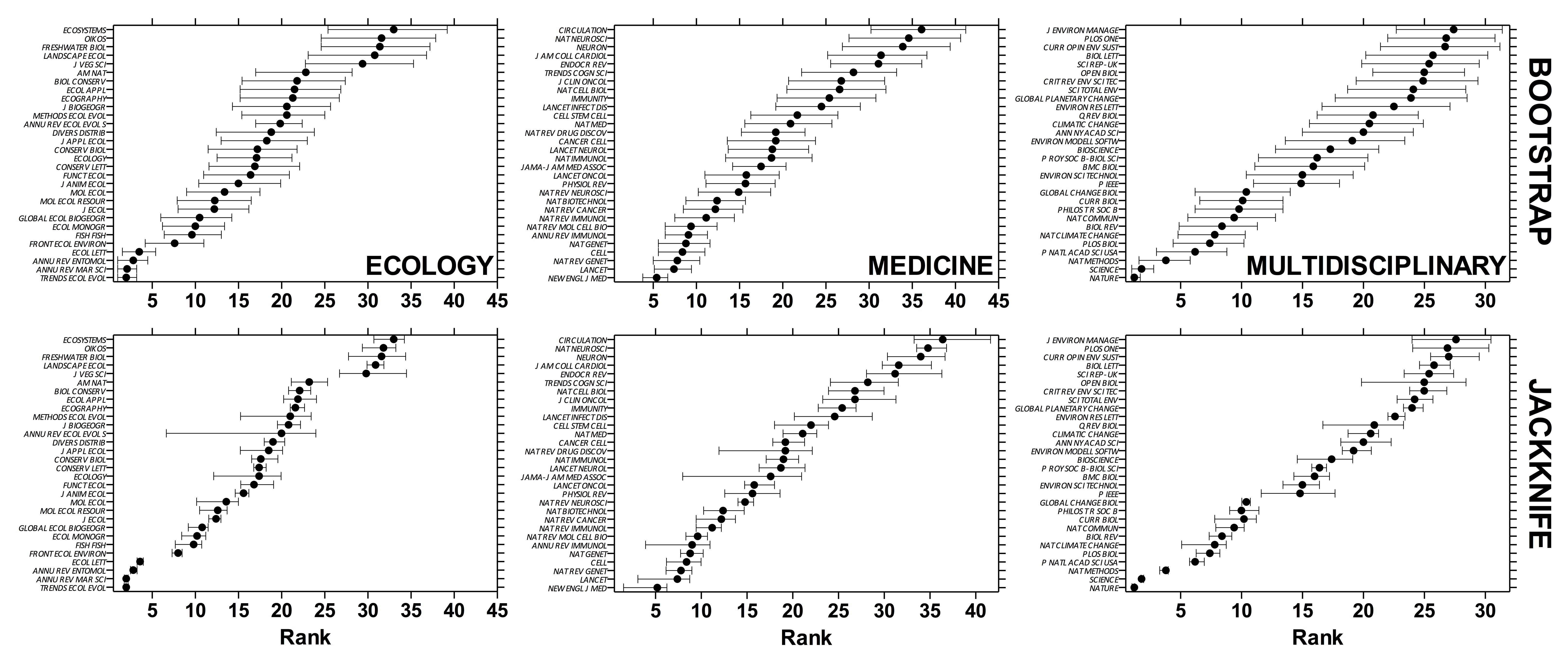

Supplement: S3 Fig — Resampled (upper panels) versus jackknife (bottom panels) for each of three journal samples (Ecology, Medicine and Multidisciplinary). (TIFF) [file pone.0149852.s005.tiff]

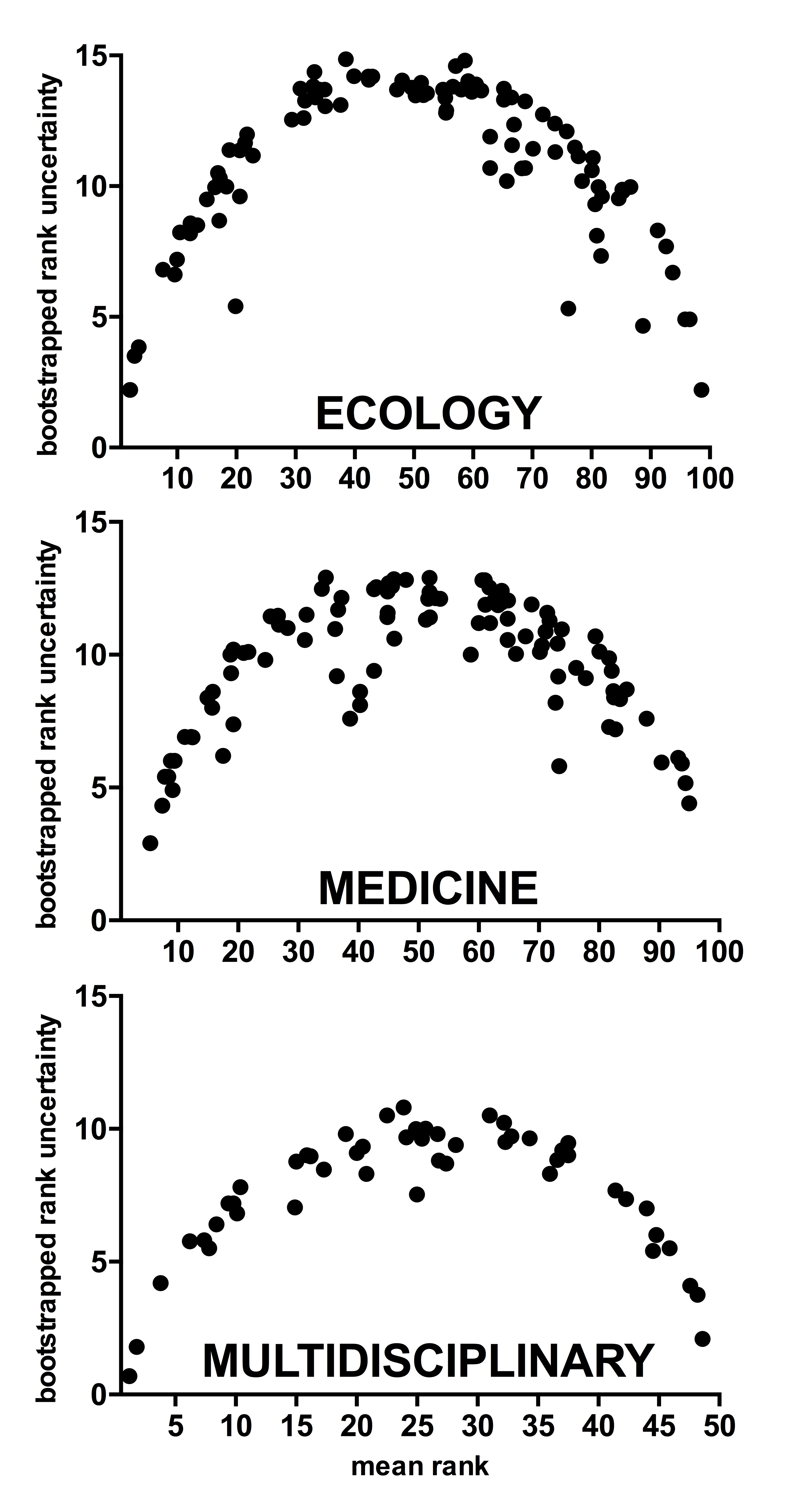

Supplement: S4 Fig — Rank uncertainty increases nonlinearly through to approximately halfway through the sample, and decrease thereafter (a characteristic of any regression fit), due to the imposed limit of 100 journals in each discipline category (50 for Multidisciplinary). Note the greater relative uncertainty for the middle ranks of the Medicine discipline. (TIFF) [file pone.0149852.s006.tiff]

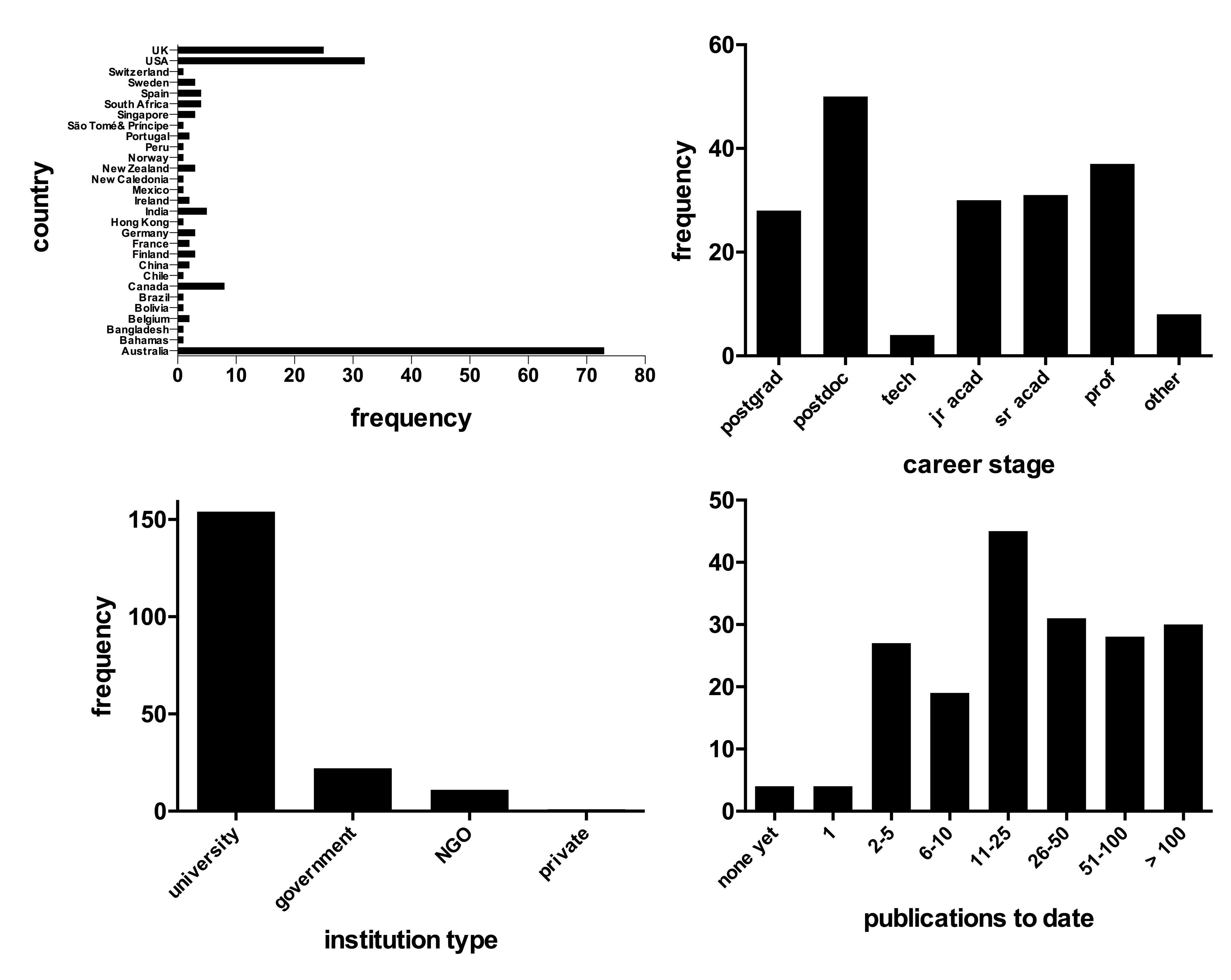

Supplement: S5 Fig — (TIFF) [file pone.0149852.s007.tiff]

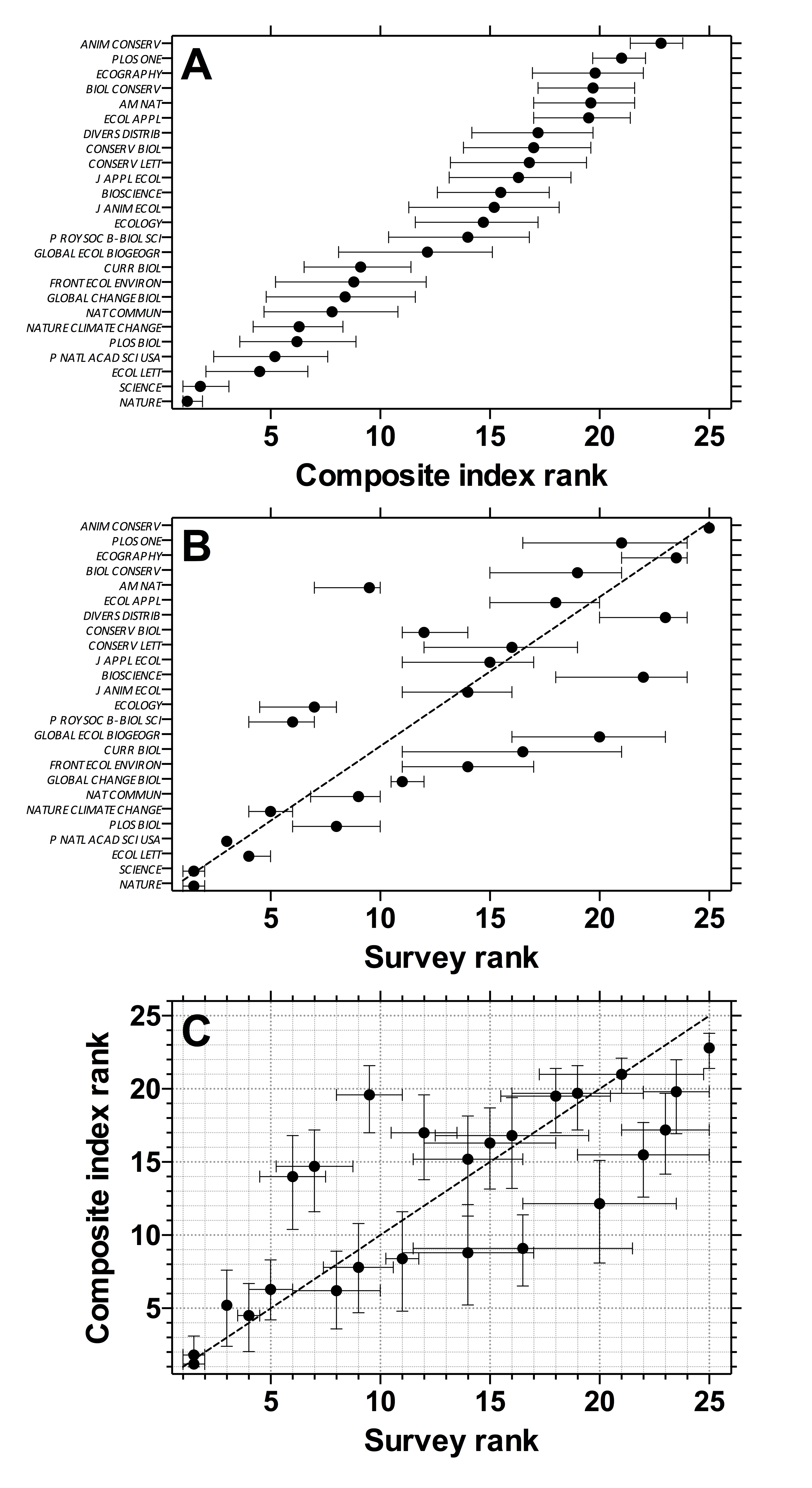

Supplement: S6 Fig — (A) Mean rank (± 95% confidence limits via κ-resampling with 10,000 iterations) of the top 25 journals within a combined Ecology and Multidisciplinary theme. Journals are ordered by mean rank of five metrics: IF, IM, SNIP, SJR and h5/log10(n) (see main text for details). (B) Mean rank (± 1σ) of the same journals assessed from a survey of 58 ecologists who had each published ≥ 50 articles. Journals above the 1:1 correspondence (45° line) are rated higher by these ecologists than their mean metric would indicate, and vice versa. (C) Overall, there was a Spearman’s rank correlation of 0.67–0.83 (compared to 0.68–0.84 for the full 188 survey participants); median = 0.76; based on 1,000 random uniform resamples of the rank interval) between both rankings. Journal abbreviations follow the Web of Science standard. (TIFF) [file pone.0149852.s008.tiff]

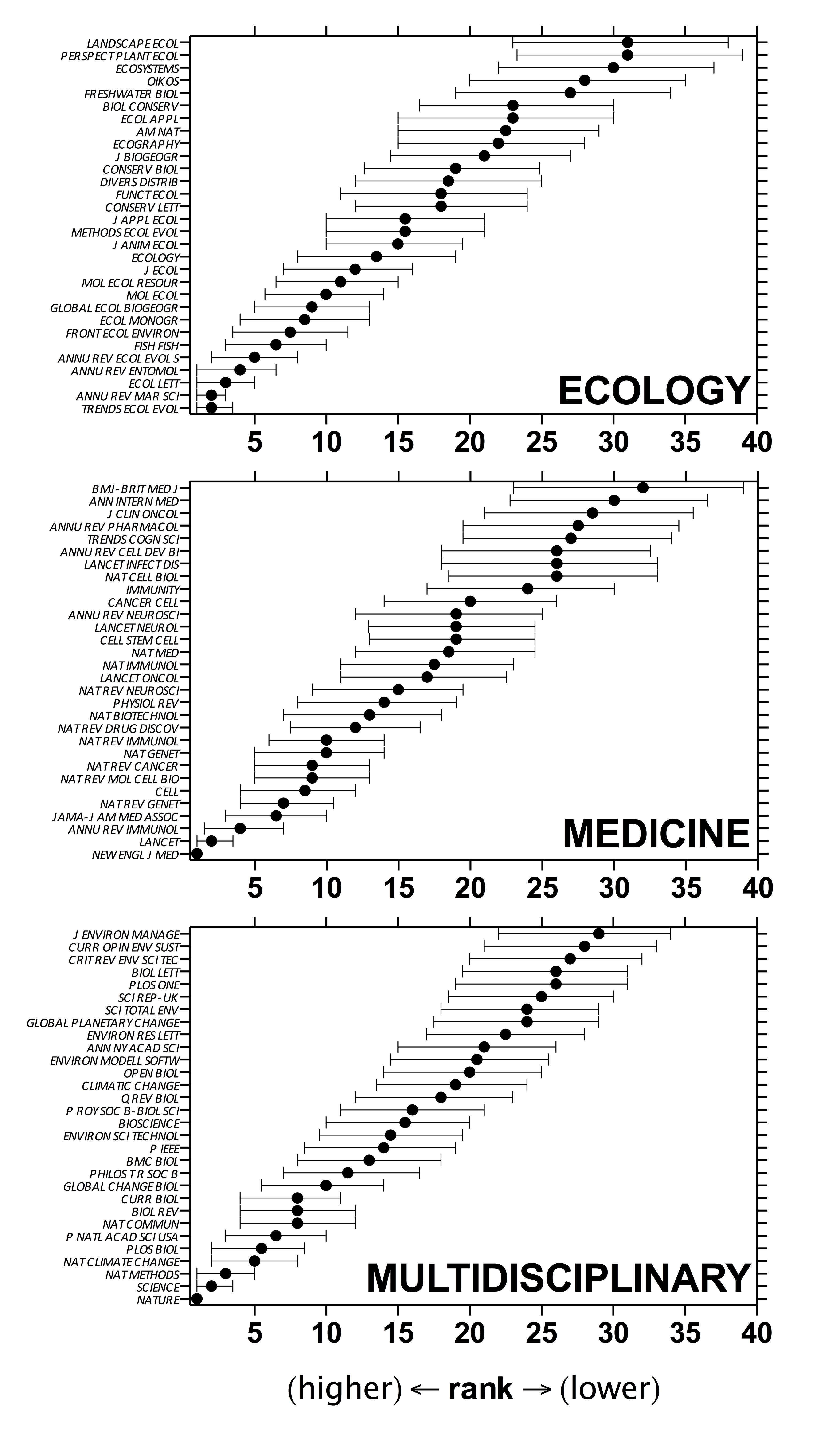

Supplement: S7 Fig — Journals are ordered by median rank of five metrics: IF, IM, SNIP, SJR and h5/log10(n); statistics were estimated using κ-resampling with 10,000 iterations, from a total sample of 100 journals for Ecology and Medicine and 50 journals for Multidisciplinary (see main text for details). Journal abbreviations follow the Web of Science standard. (TIFF) [file pone.0149852.s009.tiff]

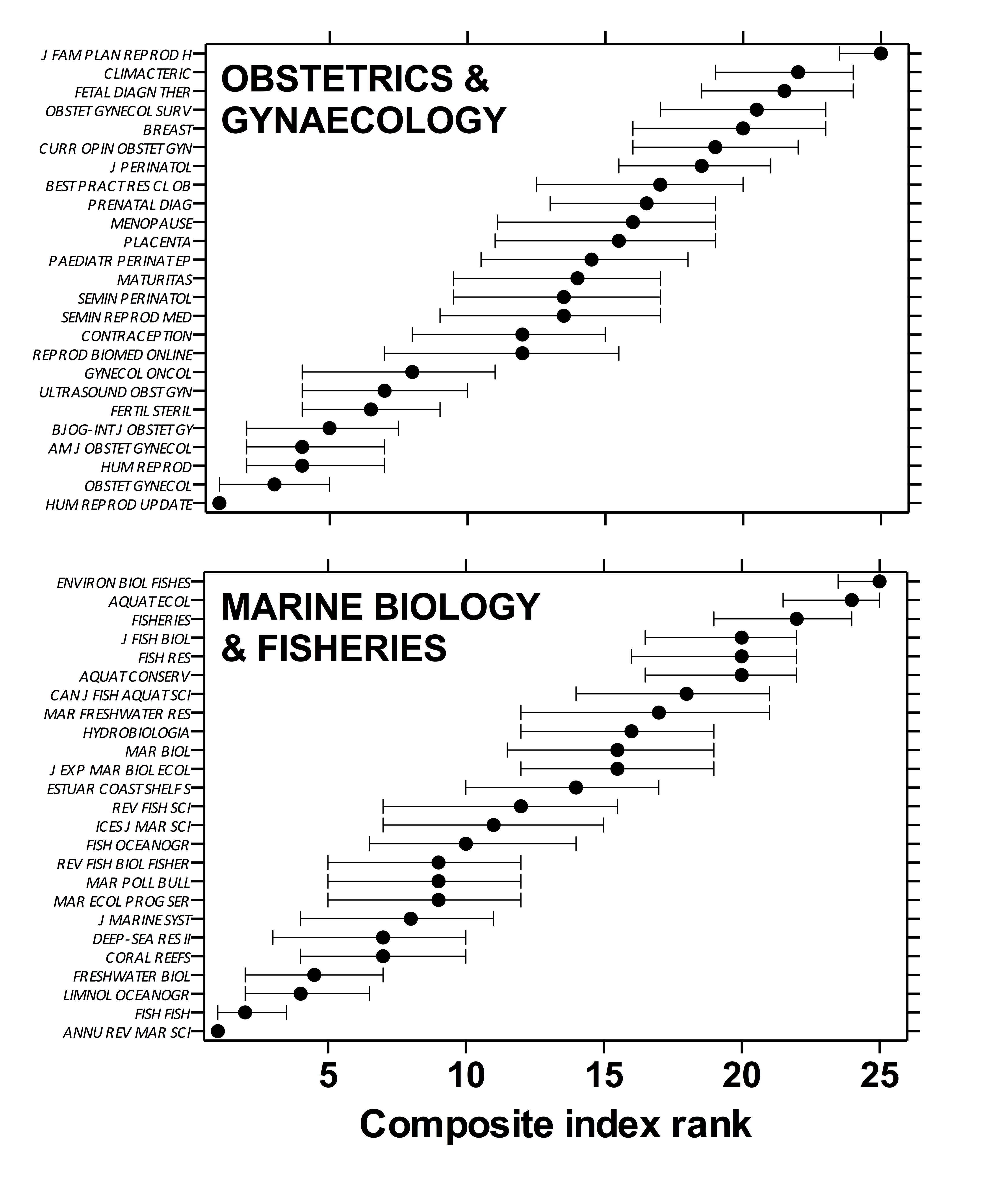

Supplement: S8 Fig — Journals are ordered by median rank of five metrics: IF, IM, SNIP, SJR and h5/log10(n); statistics were estimated using κ-resampling with 10,000 iterations (see main text for details). Journal abbreviations follow the Web of Science standard. (TIFF) [file pone.0149852.s010.tiff]

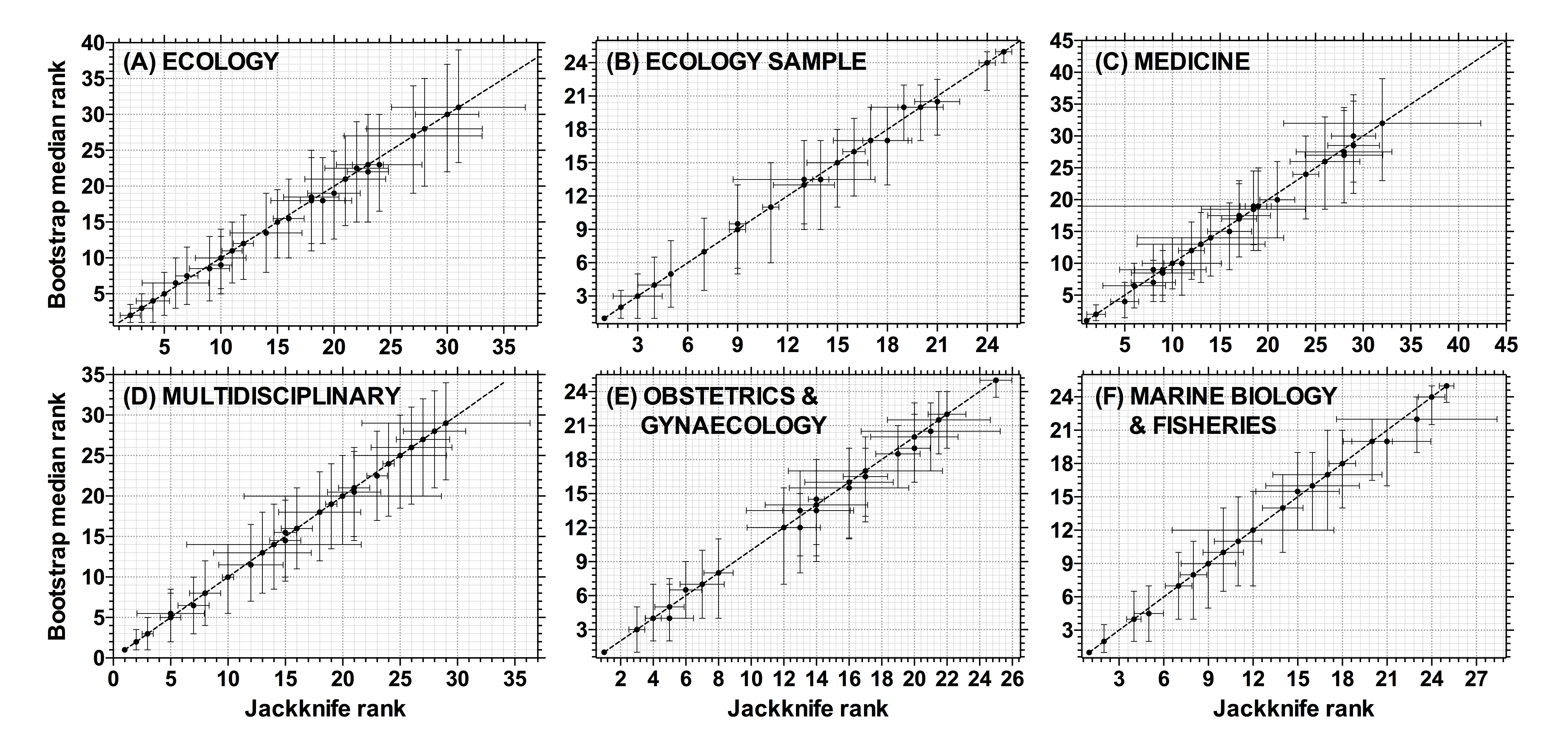

Supplement: S9 Fig — Samples include (A) Ecology, (B) Ecology Sample (Ecology and some Multidisciplinary journals), (C) Medicine, (D) Multidisciplinary, (E) Obstetrics & Gynaecology, and (F) Marine Biology & Fisheries. (TIFF) [file pone.0149852.s011.tiff]
