# Supplementary material for: How to Rank Journals
Source: PLoS One. 2016 Mar 1;11(3):e0149852. doi: 10.1371/journal.pone.0149852 (PMC4773013; doi:10.1371/journal.pone.0149852)
Supplement: S1 Table — n = total number of articles published in Journal Citation Reports (JCR; 2013) year; cites = total number of citations to the journal in the JCR year; h5 = Google 5-year h-index; h5m = median Google 5-year h-index; IF = ISI® Impact Factor; IF5 = ISI® 5-year Impact Factor; IM = ISI® Immediacy Index; SNIP = Elsevier® Source-Normalized Impact Per Paper; IPP = Elsevier® Impact Per Publication; SJR = Elsevier® SCImago Journal Rank. (DOCX) [file pone.0149852.s012.docx]

**S1 Table**. **Spearman’s *ρ* correlation matrix for the individual metrics used to develop the composite ranking**. ***n*** = total number of articles published in Journal Citation Reports [JCR; 2013] year; ***cites*** = total number of citations to the journal in the JCR year; ***h5*** = Google 5-year h-index; ***h5m*** = median Google 5-year h-index; ***IF*** = ISI® Impact Factor; ***IF5*** = ISI® 5-year Impact Factor; ***IM*** = ISI® Immediacy Index; ***SNIP*** = Elsevier® Source-Normalized Impact Per Paper; ***IPP*** = Elsevier® Impact Per Publication; ***SJR*** = Elsevier® SCImago Journal Rank.

| **ECOLOGY** | | ***n*** | **cites** | **h5** | **h5m** | **IF** | **IF5** | **IM** | **SNIP** | **IPP** |
| --- | --- | --- | --- | --- | --- | --- | --- | --- | --- | --- |
| ***cites*** | 0.73 | |  |  |  |  |  |  |  |  |
| ***h5*** | *0.64* | | 0.84 |  |  |  |  |  |  |  |
| ***h5m*** | 0.54 | | 0.78 | **0.97** |  |  |  |  |  |  |
| ***IF*** | 0.33 | | 0.63 | 0.86 | **0.90** |  |  |  |  |  |
| ***IF5*** | 0.31 | | 0.61 | 0.86 | **0.91** | **0.98** |  |  |  |  |
| ***IM*** | 0.25 | | 0.45 | 0.65 | 0.69 | 0.76 | 0.74 |  |  |  |
| ***SNIP*** | 0.29 | | 0.59 | 0.83 | 0.87 | **0.90** | **0.91** | 0.61 |  |  |
| ***IPP*** | 0.32 | | 0.63 | 0.87 | **0.91** | **0.98** | **0.98** | 0.71 | **0.94** |  |
| ***SJR*** | 0.31 | | 0.64 | 0.86 | 0.89 | **0.96** | **0.96** | 0.73 | **0.91** | **0.97** |
|  |  | |  |  |  |  |  |  |  |  |
| **MEDICINE** | | |  |  |  |  |  |  |  |  |
| ***cites*** | 0.81 | |  |  |  |  |  |  |  |  |
| ***h5*** | *0.52* | | 0.79 |  |  |  |  |  |  |  |
| ***h5m*** | 0.30 | | 0.62 | **0.91** |  |  |  |  |  |  |
| ***IF*** | -0.60 | | -0.19 | 0.26 | 0.47 |  |  |  |  |  |
| ***IF5*** | -0.60 | | -0.18 | 0.26 | 0.48 | **0.96** |  |  |  |  |
| ***IM*** | -0.41 | | -0.08 | 0.33 | 0.49 | 0.82 | 0.80 |  |  |  |
| ***SNIP*** | -0.40 | | -0.04 | 0.32 | 0.52 | 0.83 | 0.79 | 0.71 |  |  |
| ***IPP*** | -0.49 | | -0.11 | 0.30 | 0.49 | 0.88 | 0.87 | 0.70 | 0.88 |  |
| ***SJR*** | -0.39 | | -0.06 | 0.30 | 0.46 | 0.73 | 0.76 | 0.53 | 0.65 | 0.89 |
|  |  | |  |  |  |  |  |  |  |  |
| **MULTIDISCIPLINARY** | | | |  |  |  |  |  |  |  |
| ***cites*** | 0.79 | |  |  |  |  |  |  |  |  |
| ***h5*** | 0.75 | | **0.91** |  |  |  |  |  |  |  |
| ***h5m*** | 0.70 | | 0.89 | **0.99** |  |  |  |  |  |  |
| ***IF*** | 0.43 | | 0.66 | 0.83 | 0.83 |  |  |  |  |  |
| ***IF5*** | 0.40 | | 0.65 | 0.82 | 0.83 | **0.99** |  |  |  |  |
| ***IM*** | 0.37 | | 0.55 | 0.70 | 0.68 | 0.83 | 0.81 |  |  |  |
| ***SNIP*** | 0.34 | | 0.61 | 0.77 | 0.79 | 0.90 | **0.92** | 0.68 |  |  |
| ***IPP*** | 0.40 | | 0.67 | 0.84 | 0.84 | **0.98** | **0.98** | 0.81 | **0.93** |  |
| ***SJR*** | 0.42 | | 0.65 | 0.81 | 0.80 | **0.97** | **0.96** | 0.84 | 0.87 | **0.97** |
